# An Eocene shallow water isselicrinid sea lilies from the Northern Hemisphere

**DOI:** 10.1038/s41598-025-13136-7

**Published:** 2025-09-10

**Authors:** Mariusz A. Salamon, Nicolae Trif, Marcin Krajewski, Alicja Wilk, Alfonso Rubilar-Rodríguez, Bruna Poatskievick-Pierezan, Bartosz J. Płachno, Sreepat Jain

**Affiliations:** 1 Faculty of Earth Sciences, Laboratory of Palaeontology and Stratigraphy, University of Silesia in Katowice, Będzińska 60, Sosnowiec, PL-41-200 Poland; 2 Brukenthal National Museum, Natural History Museum, Cetății St. 1-550160, Sibiu, Romania; 3 Research Center for Integrated Geological Studies, 1 Mihail Kogălniceanu St, Cluj-Napoca, 4000084 Romania; 4 Faculty of Geology, Geophysics and Environmental Protection, AGH University of Krakow, al. A. Mickiewicza 30, Kraków, 30-059 Poland; 5 Sernageomin, Unidad de Paleontología y Biocronología, Santiago, Chile; 6 Technological Institute of Paleoceanography and Climate Change, itt Oceaneon, Unisinos University, São Leopoldo, Brazil; 7 Faculty of Biology, Institute of Botany, Department of Plant Cytology and Embryology, Jagiellonian University, Gronostajowa Street 9, Kraków, PL- 30-387 Poland; 8 Department of Geology, School of Applied Natural Science, Adama Science and Technology University, Adama, Oromia, ET-1888 Ethiopia

**Keywords:** Echinoderms, Crinoids, Paleogene, Eocene, Cenozoic, Europe, South America, Romania, Mesozoic marine revolution, Zoology, Ecology, Environmental sciences, Ocean sciences

## Abstract

Stalked crinoids are uncommon fossils in the Cenozoic. This is particularly due to their continuous decline starting from the Late Cretaceous and gradual restriction to the deep-sea environment, which bears a fossil record bias. On the other hand, in recent times, new data have emerged documenting some relict populations of sea lilies in the shallow marine facies from the Cenozoic. Here, we report shallow-water occurrences of Eocene crinoids from Romania that are classified as *Isselicrinus*. The representatives of *Isselicrinus* reached their greatest palaeogeographic distribution during the Eocene in offshore environments, and the only find of these crinoids from shallow-water facies was from the Southern Hemisphere. Thus, our discovery documents the first Eocene shallow-water occurrence of this taxon from the Northern Hemisphere. This finding shows that isocrinids locally might have remained in shallow environments after the initiation of the so-called Mesozoic marine revolution (MMR).

## Introduction

**Fig. 1:**
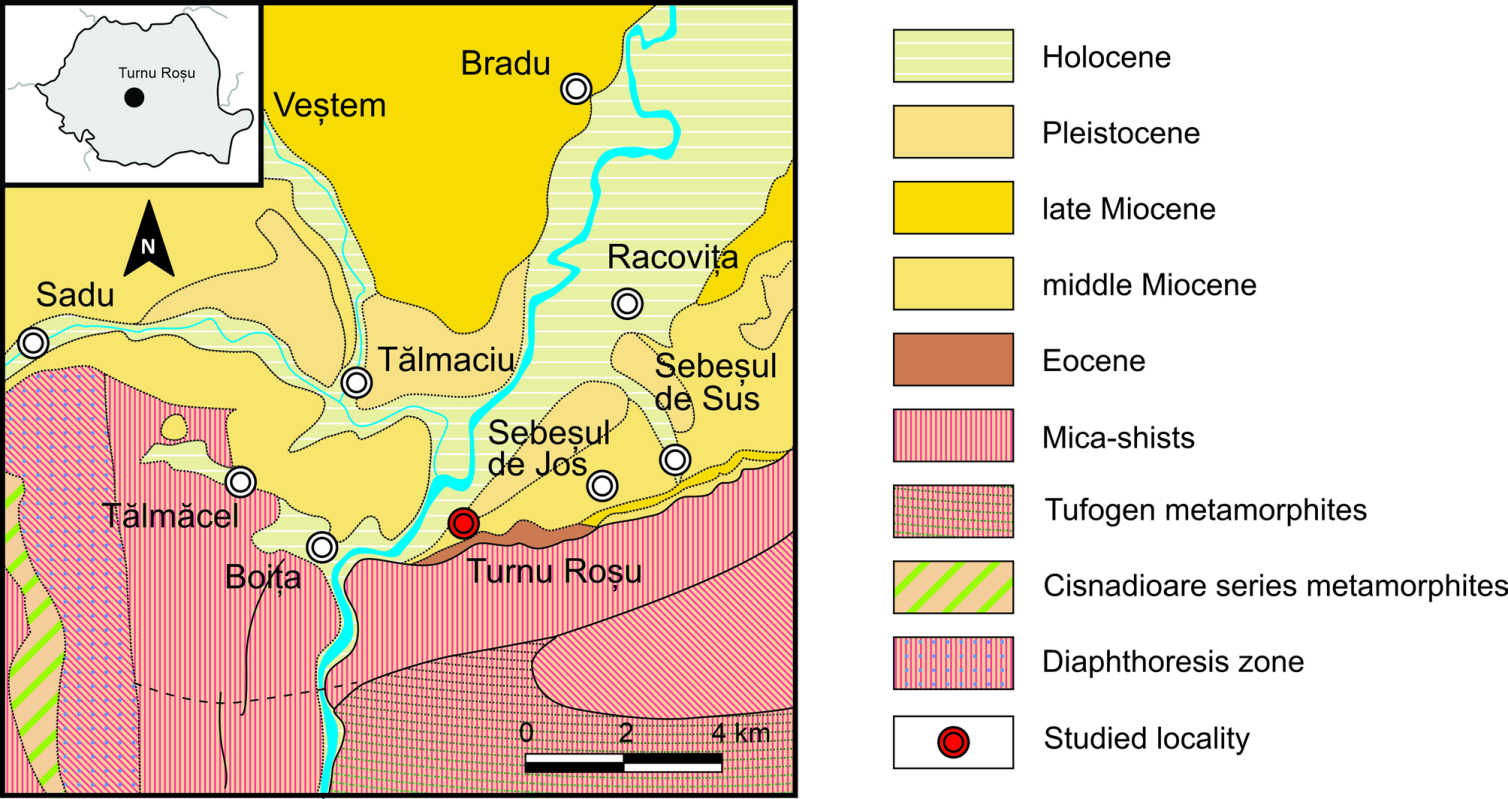
The geological map of the Turnu Roșu area (after the map of the Geological Institute of Romania, *folio* Sibiu, modified and simplified).

**Fig. 2:**
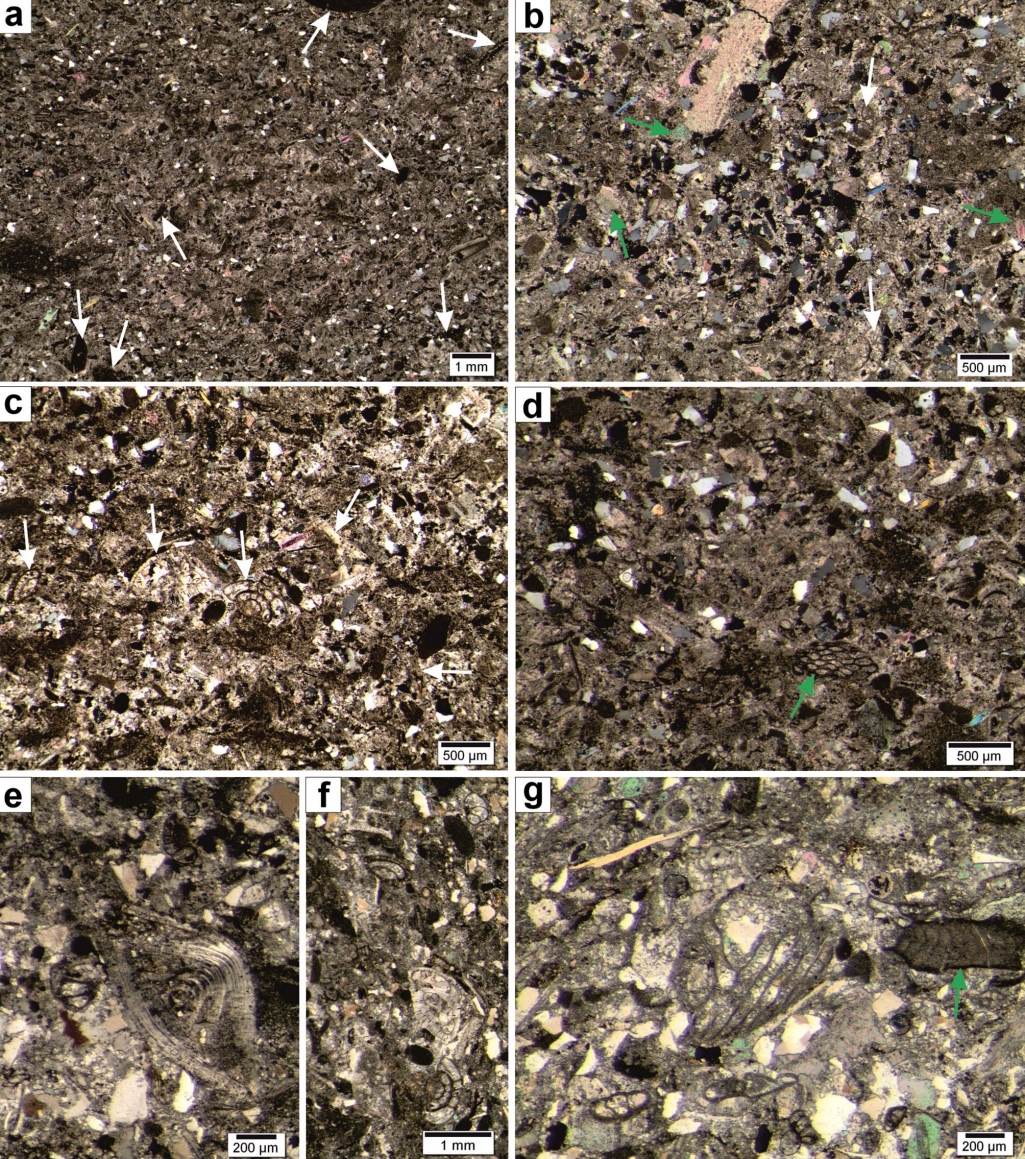
Microfacies from studied outcrop. (**a**) algal-benthic foraminifera packstone with numerous algae remains (arrows), benthic foraminifers and scattered quartz grains. (**b**,** c**) algal-benthic foraminifera packstone, locally grainstone with numerous angular quartz grains, echinoids (examples marked by green arrows) and benthic foraminifers. (**d**) fine algal-benthic foraminifera packstone/wackestone with angular quartz grains and bryozoan (green arrow). (**e-g**) examples of benthic foraminifera, algae remain (green arrow, (g)); algae-benthic foraminifera packstone.

The Paleogene fossil record of isocrinids is fragmentary, and mostly confined to deep-sea sedimentary facies. This, in part, causes some problems of taxonomic classification of post-Mesozoic isocrinids. Crinoid family Isselicrinidae was distinguished by Klikushin^1^ who indicated the presence of cryptosyzygy between primibrachials 1 and 2, and secundibrachials 1 and 2 in representatives of the subfamily Isselicrininae Klikushin, to which in the revised *Treatise* Hess and Messing^2^ included the following genera: *Austinocrinus* de Loriol, *Doreckicrinus* Rasmussen, *Isselicrinus* Roverto, and *Praeisselicrinus* Klikushin. Roux et al.^3 ^however, used the classification of isocrinids (Isocrinida Sieverts-Doreck) revised by Améziane et al.^4^ taking into account both the latest results of molecular phylogeny on extant taxa and the results of analyses derived from morphological characters, and decided to include Isselicrininae in Balanocrinidae Roux, though all other authors included this taxon in Isocrinidae Gislén (for review see Hess and Messing^2)^. Roux et al.^3^ cited, among others, the observations of Bather^5 ^who was the first to identify among isocrinids a group of “alternicirrate *Balanocrinus*” which corresponds to subfamily Isselicrininae *sensu* Améziane et al.^4^. The latter authors included in Isselicrininae, the following genera: *Praeisselicrinus* Klikushin, *Isselicrinus*, and *Panglaocrinus* Améziane, Eléaume and Roux. Hess and Messing^2^ also noted some similarities within the articular facets of *Isselicrinus* and *Balanocrinus* Agassiz (e.g., uniform marginal crenulae with adradial ridges or ribbons of minor crenulae or granules), although they decided to leave the latter within the subfamily Balanocrininae Roux (Family Isocrinidae Gislén), arguing that some specimens of *Balanocrinus*, especially those with small or pentalobate columns, have a more or less gradual transition from marginal to adradial crenulae similar to *Isocrinus*. Hess and Messing^2^ also assigned subfamily Metacrininae Klikushin to Isselicrinidae, the representatives of which had cryptosyzygy or synarthry between primibrachials 1 and 2, and the muscular articulation between secundibrachials 1 and 2. Klikushin^1^ assigned the following genera to this subfamily: *Diplocrinus* Döderlein, *Metacrinus* Carpenter, *Saracrinus* A.H. Clark, and *Teliocrinus* Döderlein, but later Klikushin^6^ included *Cenocrinus* Thomson and *Nielsenicrinus* Rassmusen, and removed *Diplocrinus* and *Teliocrinus* from this subfamily. Roux^7^ included only *Metacrinus* and *Saracrinus* in Metacrininae, i.e., forms with more than two primibrachials. Hess and Messing^2^ considered that *Eometacrinus* Baumiller and Gaździcki is also similar to *Metacrinus* and *Saracrinus* in possessing five primbrachials and muscular articulation between secundibrachials 1 and 2 and, thereof decided to include it in Metacrininae. At the same time, the authors noted that *Eometacrinus* differs from *Metacrinus* and *Saracrinus* in having the synarthrial articulation between primibrachials 1 and 2^2^. Roux et al.^8^ included *Doreckicrinus*, *Endoxocrinus* A.H. Clark (with *Endoxocrinus* and *Diplocrinus* as subgenera), *Nielsenicrinus* and *Teliocrinus* to subfamily Diplocrininae also belonging to Isselicrinidae. Representatives of this subfamily have brachitaxes typically with fewer than 5 brachials beyond secundaxil. According to Hess and Messing^2 ^Diplocrininae should include only *Cenocrinus*, *Endoxocrinus*, and *Diplocrinus*. *Nielsenicrinus* and *Teliocrinus* were included in the family Cainocrinidae Simms. Representatives of these genera are characterized by cryptosyzygy or synostosis between primibrachials 1 and 2, flat synarthry between secundibrachials 1 and 2, and cryptosyzygy or symmorphy between secundibrachials 3 and 4. The same researchers included in the Isselicrinidae the uncertain family with one genus (*Denticrinus* Klikushin), which crown remains unknown, and which have characteristic columnal facets covered by long, thick, peripheral crenulae almost perpendicular to the edge of the facet (see^9 ^text-Fig. 108; Table 10; Figs. 1 and 2).

In this paper, we describe isocrinid pluricolumnals from the Eocene of Romania assigned to *Isselicrinus*. The presented crinoids lived in a shallow marine environment and this is the first occurrence of isselicrinids in such Eocene environments within the Northern Hemisphere. This find constitutes the youngest record of shallow-sea *Isselicrinus* in the Northern Hemisphere and supports the view that some relict populations of stalked crinoids locally remained in shallow marine environments long after the initiation of the Mesozoic marine revolution.

## Geological settings and stratigraphy

The investigated specimens were collected from the locality Turnu Roșu (45°38’11.13"N, 24°17’58.46"E), situated in the south side of the Transylvanian Basin, a well-documented post-Cenomanian sedimentary basin in central Romania (Fig. 1). The Transylvanian Basin is a well-documented post-Cenomanian sedimentary basin situated in central Romania. This basin developed upon a heterogeneous foundation comprising Paleozoic crystalline units, volcanic sequences, and sedimentary rocks spanning the Triassic to mid-Cretaceous interval^10–13^. The stratigraphic architecture of the basin is characterized by four distinct tectonostratigraphic megasequences: an Upper Cretaceous, a Paleogene, a lower Miocene, and a middle to upper Miocene megasequence^13^. The investigated sediments belong to the Paleogene depositional megasequence of this basin. The deposits of this sequence exhibit excellent exposure and preservation in the northwestern sector of the basin. In contrast, the western and southern margins have a highly fragmented distribution of these deposits, with only limited exposures preserved. In the eastern sector, no Paleogene deposits are preserved.

The sedimentary succession exposed at Turnu Roșu is assigned to the Eocene. It comprises an interbedded sequence of siliciclastic, siliciclastic-carbonate, and carbonate lithofacies deposited on top of crystalline schist formations (Fig. 1). Previous investigations have documented the presence of the entire Eocene sequence within this locality^14–20^. However, strong discrepancies exist among these studies regarding the precise boundaries between the Eocene substages within this succession, thus, precluding a definitive age assignment to the collected specimens.

## Results

### Palaeoenvironment

To reconstruct a comprehensive palaeoecological framework for the Eocene epoch at the Turnu Roșu locality, an integrative approach utilizing palaeontological data, particularly from fossil vertebrates, was necessary. The most intensively studied fossil assemblages from this site are those of fish, in particular belonging to Elasmobranchii^21,22^. Following a considerable hiatus in research, the study of fossil fish at this locality was reinvigorated during the late 20th and early 21st centuries, with a series of articles that significantly advanced the taxonomic understanding of these vertebrates^23–32^. The documented presence of a diverse fish fauna within these sedimentary deposits provides a valuable foundation for inferring the ecological characteristics of the palaeoenvironments inhabited by these organisms. The ichthyofauna is composed of five types of fish assemblages: (1) small to medium littoral neritic sharks belonging to Orectolobiformes, like *Nebrius* and *Ginglymostoma*, relatively abundant in some stratigraphic levels, with a diet composed of cephalopods, bivalves, crustaceans, sea urchins, and bony fish. The extant species of *Ginglymostoma* and *Nebrius* have a benthic distribution, occupying continental and insular shelves in tropical marine environments. These elasmobranchs are frequently encountered in seagrass meadows, on sandy substrates and in proximity to coral reef formations, inhabiting depths ranging from the intertidal zone to approximately 70 m. The extant representatives of these genera have a circumglobal distribution in subtropical and tropical continental and insular waters, usually close inshore and sometimes in water deep enough only to cover them^33^; (2) small to medium sublittoral neritic sharks like *Abdounia*, *Physogaleus*, and *Rhyzoprionodon*, *Negaprion*, *Galeocerdo*, and bony fish like the Diodontidae. These sharks are encountered in the sublittoral zone feeding primarily on smaller bony fish but also on cephalopods, gastropods, and shrimps while the Diodontidae preyed on gastropods, sea-urchins, and crabs. All the above enumerated taxa prefer warm-temperate and tropical waters^34–36^; (3) medium to large nearshore to benthopelagic batoids like *Myliobatis* and *Aetobatus* that we also found to be present in almost all stratigraphic levels, taxa whose current representatives are known to prefer tropical and warm-temperate waters^37–39^; (4) large pelagic piscivor sharks like *Striatolamia*, *Macrorhizodus*, that probably only occasionally ventured near the coastline; these extinct genera, considered to be pelagic species, were likely less sensitive to water depth and temperature fluctuations. However, within the Transylvanian Basin, their fossil remains have been discovered until now in sedimentary deposits indicative of shallow-water, shoal-like conditions within the inner portions of a carbonate shelf characterized by normal salinity but also in the context of waters with greater variations in salinity but still in shallow marine settings of 40–60 m depths^40,41^; (5) apex predators with a diet that included large marine vertebrates, giant sharks belonging to the genus *Otodus*, associated with numerous ribs of sirenians, large marine herbivorous mammals that primarily consume seagrasses^42^.

The ichthyofauna counted above suggests a warm, shallow, probably tropical marine palaeoenvironment, likely a carbonate shelf with varying depths but also some areas of seagrass meadows with sandy substrates. The rest of the fossil fauna comprises a very diverse assemblage of marine taxa, including representatives from the following clades: Rhodophyta (*Lithophyllum*, *Lithothamnium*, *Jania*, *Lithoporella*, and *Archaeolithothamnium*), Cnidaria (several unidentified taxa), Gastropoda (*Campanile*,* Ampullina*,* Cepatia*,* Velates*,* Megalocypraea*,* Rimella*,* Terebellum*,* Ficus*,* Cassidaria*), Bivalvia (*Lucina*,* Miltha*,* Corbis*,* Chama*,* Cardium*,* Crassatella*,* Chlamys*,* Spondylus*,* Ostrea*), Brachiopoda (*Gryphus*,* Terebratulina*,* Megerlia*), Echinodermata (echinoids: *Gitolampas*,* Echinolampas*,* Conoclypus*,* Clypeaster*,* Schizaster*,* Macropneustes*), Cephalopoda (*Rhyncolites*), and Decapoda (*Glypturus*, *Palaeocarpilius*)^20,29,43–48^. The presence of breccia intercalations within the sedimentary sequence, in conjunction with the occurrence of freshwater vertebrates (e.g., *Trionyx*^49^; and micromammal teeth within the marine deposits (unpublished data), also suggest a depositional environment in close proximity of the shoreline and diverse marine environment with good water circulation and oxygenation.

### Thin sections analysis

#### Facies description

**Fig. 3:**
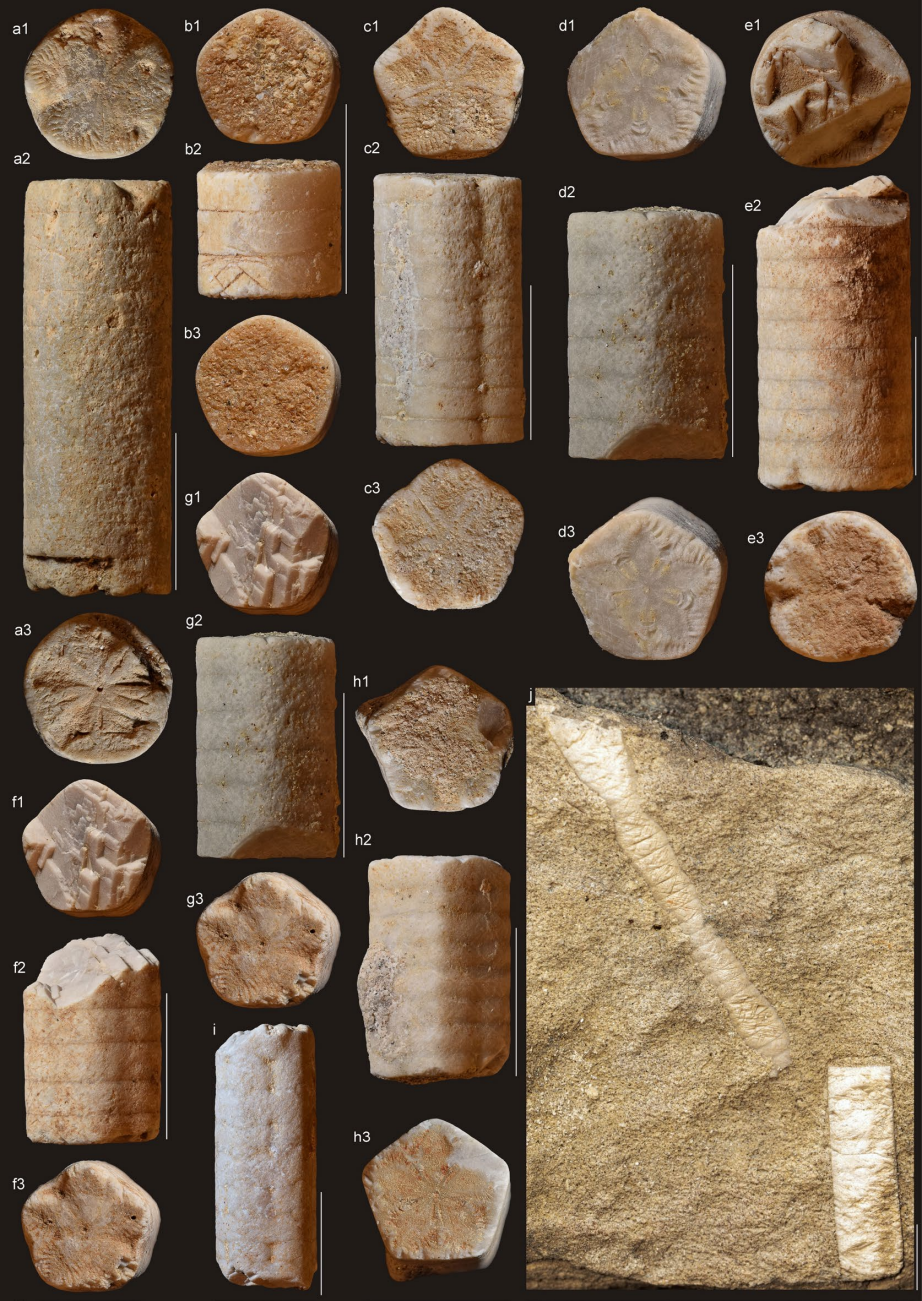
*Isselicrinus* from Eocene of Romania (Turnu Roșu area). Scale bar equals 10 mm. Acronymes: NHMS 5916–5917, 6341, 6380–6383, 39,362–39,365, and 56,408. (**a**,** e**) distal pluricolumnals (a2, e2) and columnal articular facets (a1, a3, e1, e3). (**b**,** d**) distal and/or medial pluricolumnals (b2, d2) and columnal articular facets (b1, b3, d1, d3). (**c**,** f–i**) medial pluricolumnals (c2, f2–i2) and columnal articular facets (c1, c2, f1, f3, g1, g3, h1, h3). (**j**) slab with two large stems of *Isselicrinus*; both represent the most probably distal part of stem.

The samples represent cream-coloured detrital bioclastic sandy limestone (calcarenites) with numerous well-preserved crinoid members (Figs. 2a and 3j). Macroscopic observations and microfacies analysis showed that the samples represent one facies type that can be classified as Coraline Algal-benthic foraminifera detrital sandy limestone facies (cf^50^; Fig. 2). Two microfacies types were observed within this facies: (i) dominant, algal-benthic foraminifera packstone, less often grainstone (Fig. 2b, c), and (ii) fine bioclastic wackestone (Fig. 2d). No sedimentary structures were observed in the samples, only burrows filled with fine bioclastic wackestone. The basic components of the samples are similar, although the percentage of terrigenous material in the form of dispersed quartz varies (Fig. 2b, d). Quartz grains are angular and their diameter usually ranges from 0,3 to 0,5 mm. Locally, their percentage increases significantly. The grains are dominated by numerous bioclasts, forming a grain-supported deposit with a micritic matrix. The bioclasts are dominated by numerous small remains of coralline algae (Fig. 2a-g; e.g., *Lithothamnion*) and dominantly small, less often large benthic foraminifera. Algae remains are scattered throughout the sediment and their diameter rarely exceeds 2 mm. They are badly preserved, making taxonomic identification impossible. Very numerous small benthic foraminifers (rotaliids, textulariids, miliolids) and nummulitids (Fig. 2c, e-g; cf^51,52^), can be observed. Bryozoans, echinoid spines and plates, molluscs, thin-shelled bivalves, peloids, and undifferentiated shell debris are common (Fig. 2a-g) while a subordinate amount of planktonic foraminifera can also be observed (globigerinids).

#### Facies interpretation

The sediments represent shallow-water platform deposits with an intense supply of terrigenous material from the nearby land. Well-preserved fragments of crinoids (long pluricolumnals) indicate a short transport and a very quick burial. The algae are preserved in the form of fine and dispersed detritus, suggesting increased environmental energy and intense redistribution of sediments. In the Eocene, facies dominated by Coraline Algal-benthic foraminifera with numerous echinoids and bryozoans are common in detrital limestone and had a wide distribution in shallow-water environmental settings^50,51,53–56^. During this epoch, most coralline algae and benthic foraminifers in grain-supported deposits were developed in normal marine, warm and open settings representing inner to proximal/middle mid-ramp settings. In such environments, there was an intensive redistribution of grains by waves and currents deposited in shoal conditions. Due to the rapid rate of deposition of the grain-supported loose material, these types of facies most often formed an unstable firm-type substrate. Furthermore, the presence of fine-grained angular quartz suggests rather short transport and locations close to a source area.

#### Systematic palaeontology

Systematic follows Améziane et al.^4^; description and terminology is after Hess and Messing^2^.

Order Isocrinida Sieverts-Doreck, in Moore et al.^57^.

Family Balanocrinidae Roux^7^.

Subfamily Isselicrininae Klikushin^1^.

Genus *Isselicrinus* Rovereto^58^.

Type species: *Isselicrinus insculptus* Rovereto^58^.

#### Isselicrinus sp.

*Material.* Slab with two pluricolumnals and 15 isolated pluricolumnals stored within the palaeontological collection of the Natural History Museum of Sibiu, Romania (inventory numbers NHMS 5916–5917, 6341, 6380–6383, 39362–39365, 56408). For comparative purposes additional material, represented by two slabs with columnals from collection of the Unidad de Paleontología y Biocronología, Santiago, Chile (SNGM–2032, 2033), was investigated.

*Measurements.* Pluricolumnal length varies from 7 mm to 68 mm; columnal diameter varies from 8 mm to 10,9 mm.

**Fig. 4:**
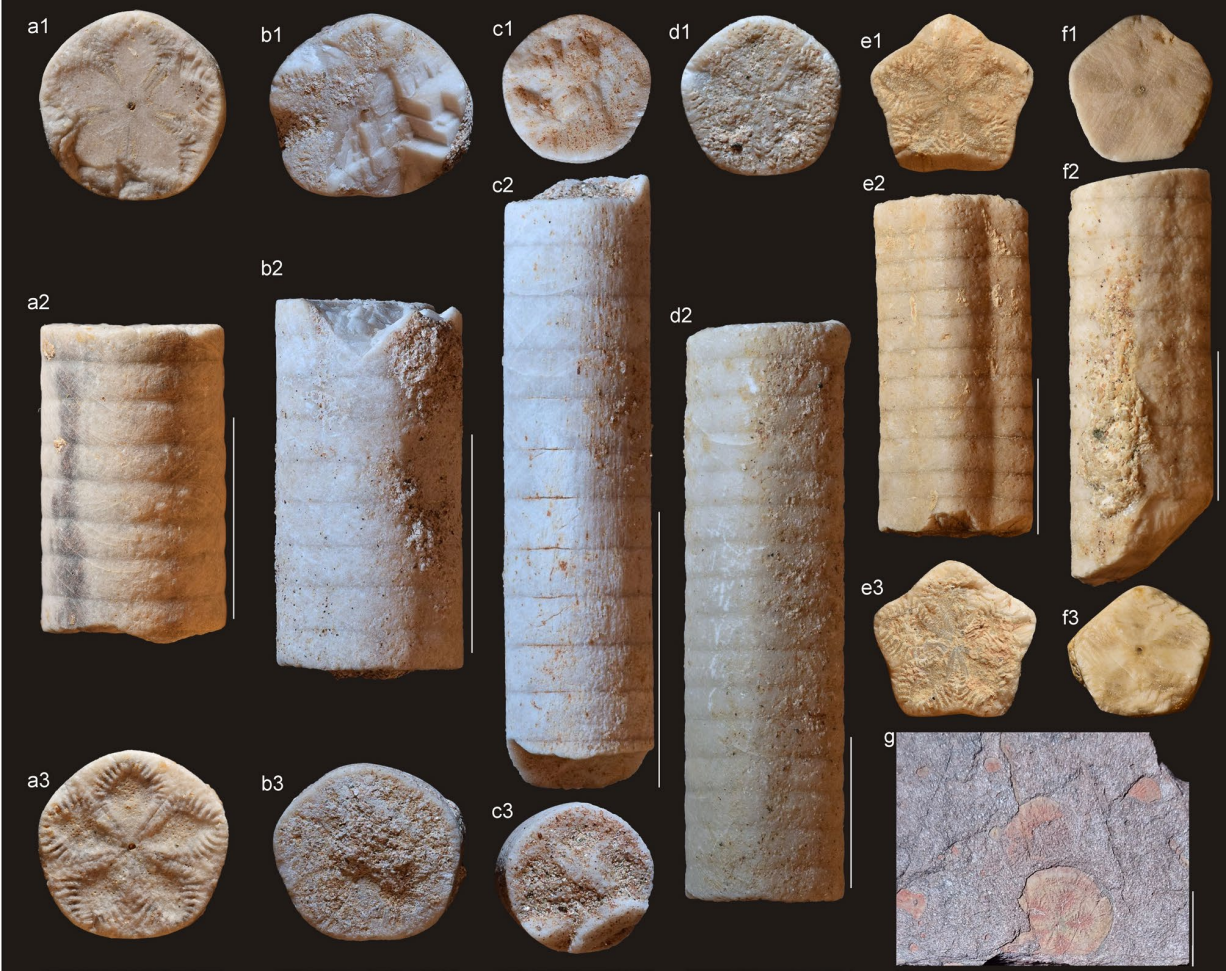
*Isselicrinus* from Eocene of Romania (Turnu Roșu area; a–f) and probably from Cretaceous of Chile (Alto del Carmen locality; g). Scale bar equals 10 mm. Acronymes: NHMS 5916–5917, 6341, 6380–6383, 39,362–39,365, 56,408 for Romanian specimens, and SNGM–2032 for Chilean individual. (**a–d**,** f**) distal and distal/medial pluricolumnals (a2, b2, c2, d2, f2) and columnal articular facets (a1, a3, b1, b3, c1, c3, d1, f1, f3). (**e**) medial/proximal pluricolumnal (e2) and columnal articular facets (e1, e2). **(g)** probably distal columnals.

*Description.* Distal columnals are cylindrical; they are pentalobate, pentagonal or stellate in case of medial and proximal columnals. Facets are covered with uniform marginal crenulae and with adradial ridges or ribbons of minor crenulae, less often granules. Petal floors are distinct and drop shaped. Cirrus sockets are small to medium sized and always facing downward from lower edge of nodal. Nodals and internodals are of the same size (Figs. 3 and 4).

*Remarks.* Complete crowns among *Isselicrinus* are known only from the early Eocene *I*. *subbasaltiformis* (Miller) from the Boreal Province^6,59^. Rasmussen^60^ indicated the presence of several species of *Isselicrinus* in Maastrichtian–Miocene sediments worldwide, while Klikushin^6^, p. 123–125; see also references to Table 1), who revised them, reported 16 species. Oji^61^ and Jagt^62^ included Cretaceous *Buchicrinus buchii* (Roemer) to *Isselicrinus*. Hess and Messing^2^ also considered the latter to be a representative of *Isselicrinus*. Roux et al.^3^ supplemented this list with one newly established species (for details see Table 1). On the other hand, Roux et al.^3^ emphasized that individual species of *Isselicrinus* are distinguished based on differences in stem structure. These authors underlined that these differences may correspond to either intraspecific changes or ecophenotypic convergence.

**Table 1:** List of *Isselicrinus* species with data on their stratigraphic and geographic distribution; data after^1–3,6,58–60,62–64,66–68,71,72,77,86–108^; plus present investigations.

Species	Stratigraphic distribution	Palaeogeographic distribution
*Isselicrinus ariakensis* (Yokoyama, 1911, sub *Pentacrinus*)^63^	Oligocene	Japan, Russia (Kamchatka)
*Isselicrinnus bermudezi* Moore and Jeffords, 1968^64^	Eocene	Cuba
*Isselicrinus cubensis* (Valette, in Riog, 1926, sub *Balanocrinus*)^65^	Miocene	Cuba
*Isselicrinus dallonii* (Termier and Termier, 1949, sub *Balanocrinus*)^66^	Oligocene	Algeria
*Isselicrinus diaboli* (Bayan, 1870, sub *Pentacrinus*)^67^	Eocene	Italy, Russia
*Isselicrinus didactylus* (d’Orbigny, in Archiac, 1846^68^, sub *Pentacrinites*, = *Pentacrinus pratti* Austin and Austin, 1846^69^ , = *Pentacrinus subbasaltiformis subrotundus* Gregorio, 1894^70^ , = *Isselicrinus insculptus* Roverto, 1914^58^)	Eocene	Austria, France, Germany, Hungary, Italy, Russia and its former republics, Spain, Switzerland; it is probable that *I*. cf. *didactylus* occurs in Eocene of Bulgaria and Israel
*Isselicrinus flamandi* (Pomel, 1887, sub *Pentacrinus*)^71^	Eocene	Algeria
*Isselicrinus haitiensis* (Springer, 1924, sub *Balanocrinus*)^72^	Miocene	Haiti
*Isselicrinus lorioli* (Noelli, 1900^73^ , sub *Pentacrinus*; non *Pentacrinus lorioli* Woodward, 1894^74^ , nom. nudum)	Miocene	Italy
*Isselicrinus pellegrinii* (Meneghini, 1876^75^, sub *Pentacrinus*; = *Pentacrinus inkermanensis* de Loriol, 1877^76^ ; = *Pentacrinus taramellii* Marioni, in Dainelli, 1915^77^)	Eocene	Italy, Russia and its former republics
*Isselicrinus perredoni* (Pomel, 1887, sub *Pentacrinus*)^71^	Oligocene	Algeria
*Isselicrinus rotularis* (Guppy, 1874, sub *Pentacrinus*; = *Pentacrinus obtusus* Guppy, 1874)^78^	Eocene	Trinidad
*Isselicrinus subbasaltiformis* (Miller, 1821^78^ , sub *Pentacrinites*; = “*Pentacrinus*” *fraasi* Biese, 1935^80^)	Eocene	Denmark, England, Germany
*Isselicrinus paucicirrhus* (Nielsen, 1913)^81^	Paleocene	Denmark
*Isselicrinus sulcifer* (Eichwad, 1871, sub *Pentacrinus*)^82^	Eocene	Russia and its former republics
*Isselicrinus sundaicus* (Wanner, 1938^83^ , sub *Balanocrinus*; = *Balanocrinus* sp.: Sieverts, 1932^84^)	Miocene	Indonesia and surrounding areas
*Isselicrinus baldoensis* Roux, Martinez-Soares, Fornaciari, Gatto, Papazzoni and Giusberti, 2024^3^	Eocene	Italy
*Isselicrinus buchii* (Roemer); = *Buchicrinus buchii* (Roemer)	Maastrichtian	Algeria, Egypt, England, France, Germany, Greenland, France, Netherlands, Russia and its former republics, Sweden, Tunisia, Ukraine
*Isselicrinus* sp. 1	probably Late Cretaceous	Northern Chile (Fig. 4g)
*Isselicrinus* sp. 2	Eocene	Antarctica, Greenland, Romania, USA, former Yugoslavia, southern Chile and Argentina (Tierra del Fuego)

**Fig. 5:**
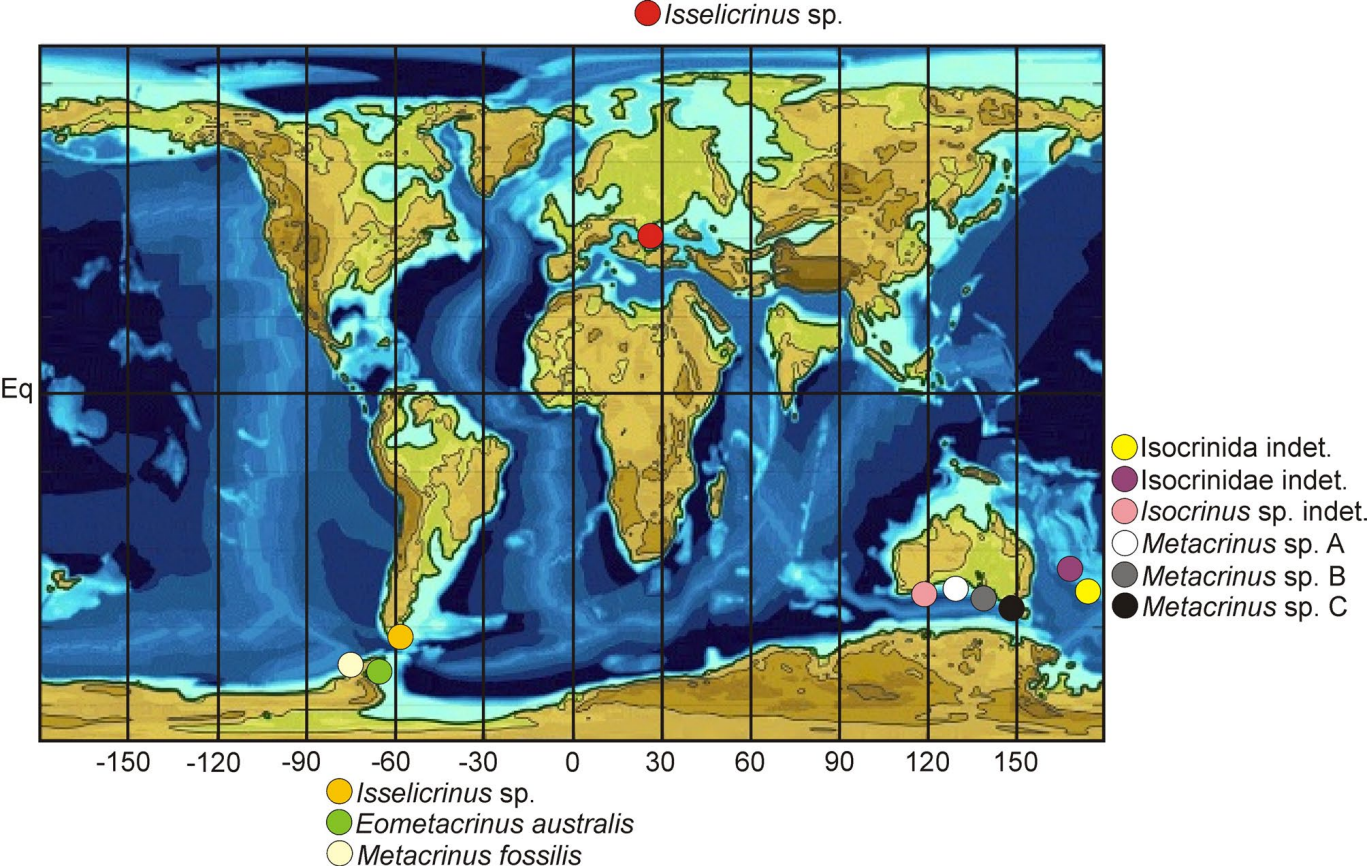
Palaeogeographic distribution of shallow-water Eocene isocrinids (data taken from^111^ and literature cited therein, plus current study). Map after^132^slightly modified).

For stratigraphic and palaeogeographic distrubution see Table 1; Fig. 5.

## Discussion

Roux et al.^3^ and literature cited therein) pointed out that *Isselicrinus* reached its greatest palaeogeographic distribution during the Eocene in offshore environments, especially on upper bathyal slopes^106,108^. Only one *Isselicrinus* Eocene occurrence was so far recorded from shallow-water environments. Malumián and Olivero^109^ described *Isselicrinus* from Tierra del Fuego Island (South America) and stated that fossil crinoids from Tierra del Fuego Island are all isocrinids restricted to the shallow-water Leticia Formation of late middle Eocene age (see also^110^). The remaining shallow-water Eocenian isocrinids have been also documented within the Southern Hemisphere. In the case of Australia and Oceania, these were: Isocrinida indet., Isocrindiae indet., *Metacrinus* sp. 1, 2, and 3, and in Antarctica: *Eometacrinus australis* Baumiller and Gaździcki, and *Metacrinus fossilis* Rasmussen (see Fig. 1 in^111 ^and references cited therein); Fig. 5. Thus, our find constitutes the first Eocene shallow-water occurrence of *Isselicrinus* from the Northern Hemisphere.

Meyer and Macurda^112^ argued that stalked crinoids disappeared globally from shallow-water settings in the mid- to late Mesozoic due to increased predation pressure in such environments. A similar view was expressed by Bottjer and Jablonski^113 ^who quantitatively demonstrated that isocrinids became restricted to middle-shelf and deeper environments starting from the Late Cretaceous. The process of colonization of these deep environments was supposed to have ended by the Eocene. The above-mentioned authors concluded that isocrinids have retained this environmental distribution to the present. Admittedly, there are few reports of the presence of stalked crinoids in shallow-water environments in the Cenozoic (e.g., Oligocene of Germany, see^114^); Paleocene of New Jersey, see^115^. According to Oji^116^ there were at least four records of shallow-water isocrinids since the mid-Cretaceous. They were: *Metacrinus* sp. from the Paleocene deposits on South Island (New Zealand), ?*Nielsenicrinus* sp. from the late Paleocene of western Australia, *Metacrinus fossilis* from the Eocene of Seymour Island (Antarctic Peninsula), and *Nielsenicrinus* sp. from a late Oligocene of the North Island (New Zealand); however, the latter author added that the affinity of crinoids classified by Eagle^117^ as *Nielsenicrinus* is questionable. A number of studies have subsequently appeared documenting stalked crinoids from the Upper Cretaceous and post-Cretaceous shallow-sea sediments. Jagt^62^ reported occurrence of shallow-water isocrinids (including *Isselicrinus buchii*) in the Maastrichtian of Belgium and the Netherlands. Furthermore, recent discoveries of stalked forms in the late Mesozoic of Europe support the view that they were able to survive in shallow (and very shallow) settings^118–128^; see also comments in^129^. Similar results were presented by Whittle et al.^130^ (see also^110^), who based on a series of occurrences of post-Mesozoic isocrinids in shallow-water facies of the Southern Hemisphere, arguing that the MMR was not globally synchronous. Noteworthy, Salamon et al.^111^ reported the anomalous occurrence of the isocrinids in Miocene shallow nearshore marine facies from Northern Hemisphere (Central Paratethys). Globally, this was the youngest record of shallow-sea stalked crinoids. These authors concluded that some relict stalked crinoids may have been able to live in the shallow-water environments by the middle Miocene, which further confirms that the depth restriction of isocrinids to offshore environments was not synchronous globally, as first suggested by Oji^116^ and later by Whittle et al.^130^. However, the fact that some relict populations of isocrinids survived in shallow-sea environments even in the Miocene does not question the predation-related hypothesis; rather the timing and synchronicity of the migration/depth restriction are a subject of discussion. Notwithstanding the above, it must be emphasized that today stalked crinoids are only restricted to deep water settings, but in their evolutionary history they were widespread in the shallow-sea environments. Noteworthy, Oji^116^ using regenerated arms in isocrinids as a sublethal predation proxy, has demonstrated that predation intensity reduces with increasing depth. More recently, time-series data collected over several years from Recent deep-sea environment (Caribbean Sea, Roatán), have documented a low incidence of injuries, low healing rates, and low mortality of isocrinids resulting from fish predators^131 ^supporting the hypothesis that the deep-sea environments represent a predation refuge for stalked crinoids.

## Materials and methods

The specimens examined in this study are derived from two collections. The majority are housed within the paleontological collection of the Natural History Museum, Sibiu, Romania (inventory numbers NHMS 5916–5917, 6341, 6380–6383, 39362–39365, 56408) having been collected during the 19th and early 20th centuries. Two additional specimens are curated within the Palaeontology and Stratigraphy Museum of Babeș-Bolyai University in Cluj-Napoca (inventory numbers BBUPSM 24413a-b). These latter specimens were collected by one of the authors (TN) during field investigations within the Turnu Roșu locality, thereby enabling a precise provenance. Additional material is represented by two slabs with columnals, collected by the geologist E. Salazar in northern Chile (near Alto del Carmen locality), which are deposited in the Palaeontological Collection of the Servicio Nacional de Geología y Minería, Santiago, Chile (SNGM–2032, 2033; Fig. 4g). Although no direct ages have been obtained from the sampled section (first refered as ‘Pucalume Fm’), the same unit, further south, has reported U-Pb detrital zircon maximum depositional ages of ca. 92 Ma and 89 Ma, and is covered by volcanics deposits of ca. 80 Ma^133,134^.

Photographic documentation of the specimens was obtained using a Nikon D5300 camera equipped with a Sigma 105 mm lens. To enhance image clarity, image stacking method was used, with image processing conducted using Combine ZP 1.0 software.

Thin sections were prepared at the AGH University of Kraków, Poland. Thin sections were used for facies/microfacies analyses and photographed using Olympus SZX 7 and a camera Olympus SC 30, together with specialized Olympus Stream 1.7 software, housed at the AGH University of Kraków.

All figures in this article were created in CorelDraw 2021 (www.coreldraw.com; version: 23.5.0.506) purchased by the University of Silesia in Katowice, Poland.

## Data Availability

All data generated or analysed during this study are included in this published article.
